# Prenatal Exposure to Traffic Pollution and Childhood Body Mass Index Trajectory

**DOI:** 10.3389/fendo.2018.00771

**Published:** 2019-01-07

**Authors:** Abby F. Fleisch, Izzuddin M. Aris, Sheryl L. Rifas-Shiman, Brent A. Coull, Heike Luttmann-Gibson, Petros Koutrakis, Joel D. Schwartz, Itai Kloog, Diane R. Gold, Emily Oken

**Affiliations:** ^1^Pediatric Endocrinology and Diabetes, Maine Medical Center, Portland, ME, United States; ^2^Center for Outcomes Research and Evaluation, Maine Medical Center Research Institute, Portland, ME, United States; ^3^Division of Chronic Disease Research Across the Lifecourse, Department of Population Medicine, Harvard Medical School and Harvard Pilgrim Health Care Institute, Boston, MA, United States; ^4^Department of Obstetrics and Gynecology, Yong Loo Lin School of Medicine, National University of Singapore, Singapore, Singapore; ^5^Department of Biostatistics, Harvard School of Public Health, Boston, MA, United States; ^6^Department of Environmental Health, Harvard School of Public Health, Boston, MA, United States; ^7^Department of Geography and Environmental Development, Ben-Gurion University of the Negev, Beersheba, Israel; ^8^Channing Laboratory, Brigham and Women's Hospital, Boston, MA, United States; ^9^Department of Nutrition, Harvard School of Public Health, Boston, MA, United States

**Keywords:** air pollution, particulate matter, traffic, growth, childhood

## Abstract

**Background:** Limited evidence suggests an association between prenatal exposure to traffic pollution and greater adiposity in childhood, but the time window during which growth may be most affected is not known.

**Methods:** We studied 1,649 children in Project Viva, a Boston-area pre-birth cohort. We used spatiotemporal models to estimate prenatal residential air pollution exposures and geographic information systems to estimate neighborhood traffic density and roadway proximity. We used weight and stature measurements at clinical and research visits to estimate a BMI trajectory for each child with mixed-effects natural cubic spline models. In primary analyses, we examined associations of residential PM_2.5_ and black carbon (BC) exposures during the third trimester and neighborhood traffic density and home roadway proximity at birth address with (1) estimated BMI at 6 month intervals through 10 years of age, (2) magnitude and timing of BMI peak and rebound, and (3) overall BMI trajectory. In secondary analyses, we examined associations of residential PM_2.5_ and BC exposures during the first and second trimesters with BMI outcomes.

**Results:** Median (interquartile range; IQR) concentration of residential air pollution during the third trimester was 11.4 (1.7) μg/m^3^ for PM_2.5_ and 0.7 (0.3) μg/m^3^ for BC. Participants had a median (IQR) of 13 (7) clinical or research BMI measures from 0 to 10 years of age. None of the traffic pollution exposures were significantly associated with any of the BMI outcomes in covariate-adjusted models, although effect estimates were in the hypothesized direction for neighborhood traffic density and home roadway proximity. For example, greater neighborhood traffic density [median (IQR) 857 (1,452) vehicles/day x km of road within 100 m of residential address at delivery] was associated with a higher BMI throughout childhood, with the strongest associations in early childhood [e.g., per IQR increment natural log-transformed neighborhood traffic density, BMI at 12 months of age was 0.05 (−0.03, 0.13) kg/m^2^ higher and infancy peak BMI was 0.05 (−0.03, 0.14) kg/m^2^ higher].

**Conclusions:** We found no evidence for a persistent effect of prenatal exposure to traffic pollution on BMI trajectory from birth through mid-childhood in a population exposed to modest levels of air pollution.

## Introduction

Childhood obesity often tracks into adulthood (1), leading to costly comorbidities and lower life expectancy in affected individuals (2). Obesity is primarily a result of high caloric intake and low physical activity, but eating less and exercising more has proven very difficult to maintain (3). Prenatal and early life exposure to some environmental toxicants may also predispose children to obesity (4). It is a public health imperative to evaluate early life determinants of obesity and identify vulnerable windows of exposure because of the potential for preventive interventions.

In rodents, early life air pollution exposure results in systemic and adipose inflammation and leads to greater visceral adiposity (5). Consistent with this observation, epidemiologic studies have demonstrated an association between air pollution exposure during childhood and increased risk of overweight or obesity (6–10). Greater maternal air pollution exposure during pregnancy also may be associated with greater offspring adiposity and increased risk of overweight or obesity in childhood (11–15). Prenatal air pollution may affect child weight directly by increasing inflammatory potential of fetal adipose tissue or indirectly by restricting fetal growth or disrupting maternal glycemia, which have both been associated with prenatal air pollution exposure in our cohort (11, 12) and others (16, 17) and may prime children for greater weight or adiposity later in life (18, 19). In our cohort, late prenatal traffic pollution exposure was associated with greater weight gain during infancy (12), and infants of mothers living closer to a major roadway at the time of delivery had greater adiposity but no difference in BMI in mid-childhood (11). Despite the fact that obesity is more prevalent as children age (20), there has been no prior evaluation of the extent to which prenatal traffic pollution exposure may differentially impact growth at different time windows during childhood.

In the present analysis, we used a growth trajectory approach to evaluate the impact of traffic pollution exposure on critical windows of growth across childhood. Our primary objective was to evaluate the extent to which late prenatal exposures to fine particulate matter (PM_2.5_) and black carbon (BC) (a traffic-related component of PM_2.5_), as well as residential traffic density and roadway proximity, were associated with trajectory of body mass index (BMI) from birth to mid-childhood. In secondary analyses, we also evaluated the role of early and mid-prenatal exposures to traffic pollution on BMI trajectory. We hypothesized that higher prenatal traffic pollution exposure would be associated with lower BMI at birth and greater gains in BMI throughout childhood.

## Materials and Methods

### Study Population and Design

Participants were recruited during 1999–2002 to Project Viva, a prospective observational cohort study of prenatal exposures and offspring health. We recruited women during their first prenatal visit (median 10 weeks gestation) at Atrius Harvard Vanguard Medical Associates, a multi-specialty group practice in eastern Massachusetts. Children had study visits at the time of birth and in infancy (median: 6.0 months of age), early childhood (median: 3.3 years of age), and mid-childhood (median: 7.7 years of age). We have previously published details of our recruitment procedures and study protocol (21).

Of 2,128 live singleton births, we modeled BMI trajectories in 1,649 (77.5%) offspring who had data for at least 3 measurements of BMI from birth to mid-childhood (our criterion for inclusion in the growth trajectory model) and at least one air pollution exposure metric. Children included vs. excluded in this analysis were more likely to have older and more educated mothers, had a larger gestational age at delivery, and were more likely to be white or other race/ethnicity and less likely to be black, Hispanic, or Asian (Supplemental Table 1).

Mothers provided written informed consent at enrollment and for their child at each in-person visit. Project Viva has been reviewed and approved by the Institutional Review Board of Harvard Pilgrim Health Care. The present secondary data analysis was deemed exempt by the Maine Medical Center Institutional Review Board.

### Air Pollution Exposures

We used aerosol optical depth data to estimate PM_2.5_ exposure at each participant's residence at a 1 × 1 km spatial grid resolution (mean daily “out-of-sample” ten-fold cross-validation *R*^2^ = 0.88) (22). We used a land-use regression model to estimate daily BC exposure at each residence (mean “out-of-sample” 10-fold cross-validation *R*^2^ = 0.73) (23). Our PM_2.5_ model encompassed addresses in the New England region, and our BC model encompassed addresses in Eastern Massachusetts.

Because we have previously shown 3rd trimester exposure to traffic pollution to be most closely associated with fetal and infant growth in Project Viva (12), in primary analyses, we used estimates of residential traffic pollution during the 3rd trimester, which we obtained by averaging daily exposures from the 188th day (i.e.—27 weeks gestation) after the last menstrual period (LMP) to the day before birth. For secondary analyses, we used estimated average residential traffic pollution during the 1st trimester [date of LMP to 93rd day after LMP (i.e.—13 weeks gestation)] and 2nd trimester (94th day after LMP to 187th day after LMP). We assigned exposures to addresses where we had data available for at least 90% of days in the trimester. Our estimates of PM_2.5_ and BC exposure accounted for residential moves during pregnancy.

We used the 2002 road inventory from the Massachusetts Executive Office of Transportation to calculate traffic density at each participant's residential address at the time of delivery by multiplying annual average daily traffic (vehicles/day) by length of road (km) within 100 m of participants' residential address. We used 2005 ESRI Street Map™ North America ArcGIS 10 Data and Maps to estimate home roadway proximity at the time of delivery as distance to Census Feature Class Code A1 or A2 roads (i.e.—highways).

### Child Anthropometric Measures

We obtained anthropometric measures from study visits and from clinical records. At study visits, research assistants measured participants' weight using an electronic scale [Seca scale in infancy and early childhood (Hanover, MD); Tanita scale in mid-childhood (Arlington Heights, IL)] and length/height in infancy and early childhood using a Shorr measuring board and height in mid-childhood using a stadiometer (Shorr Productions, Olney, MD). We also reviewed data from each participant's pediatrician's office to obtain length/height and weight data from clinical visits during infancy and childhood. We have previously shown clinical measurements of length, obtained by the paper and pencil method, to systematically overestimate research measures by 1.8 cm in children <2 years of age (24), so we included this correction factor for clinical lengths obtained in this age group. Using both research and clinical measures, we calculated BMI as weight in kilograms divided by length or height in meters squared.

### Covariates

We collected information on maternal race/ethnicity, education, parity, and smoking habits by questionnaire at study enrollment and on child race/ethnicity by questionnaire in early childhood. We obtained child sex, birth weight, and date of birth from the hospital medical record. We calculated length of gestation by last menstrual period, and we updated it with mid-pregnancy ultrasound if the two estimates differed by >10 days. We abstracted residential census tract median annual household income and percent below poverty at the time of delivery from 2000 US Census data (25).

### Statistical Analyses

#### Modeling BMI Trajectory

We fit individual BMI curves using mixed-effects models with natural cubic spline functions for age, as previously described (26). The fixed effects component of the model was:

(1) $$\begin{array}{l} BMI=\beta_{00}+\beta_{10}(\text{age})+\sum_{j=1}^{m}\beta \text{j}\{(\text{age}-k_{\text{j}}{)}_{+}^{3}\\ \;-\lambda j{\left(\text{age}-k_{\min}\right)}_{+}^{3}-\left(1-\lambda \text{j}\right){\left(\text{age}-k_{\max}\right)}_{+}^{3}\}+e_{ij} \end{array}$$

where *k*_min_ and *k*_max_ = boundary knots, *k*_j_ = interior knot point *j* between boundary knots; *m* = number of interior knots between boundary knots; *j* = 1, 2, …, m; *e* = residual and (age-k_j_${)}_{+}^{3}$ is defined as age–k if age ≥ k_j_. We included random effects for the intercept, linear age slopes and spline functions to account for repeated measures in the same child and capture the non-linear trend in BMI. Our final model included interactions of child sex with spline terms as fixed parameters, as BMI trajectories derived were similar using this approach as compared to modeling BMI trajectories separately for boys and girls.

We considered two approaches to select knot locations: at equally spaced percentiles or at the median, minimum and maximum ages of each of three developmental periods: infancy and early and mid-childhood. We used Bayesian information criterion to determine the optimal number (six) and location (0.1, 4.9, 10.6, 37.9, 92.5, and 131.1 months of age) of knots for both fixed and random effects.

We estimated child age at peak and rebound by differentiation of the subject-specific BMI curve; the peak and rebound are located at ages where the derivative of the curve equals zero. We estimated the magnitude (kg/m^2^) at peak and rebound as the highest and lowest points, respectively, of the child-specific BMI curve. We defined velocity (kg/m^2^/month) to BMI peak as the linear velocity from birth to the BMI peak, and velocity to rebound as the linear velocity from BMI peak to rebound. We also used the modeled trajectory to predict BMI at 6-monthly intervals from birth to 10 years for each child. Among the 1,649 children, 1,578 (74.2%) had estimable BMI peak and rebound, 41 had no BMI peak (i.e., showed no decline in BMI after the rise in infancy) and 30 had no BMI rebound (i.e., showed no rise in BMI after the decline in early childhood).

#### Examining Associations of Prenatal Air Pollution Exposure With BMI Trajectory

We ran separate linear regression models to examine the associations of exposure to each air pollutant with BMI in childhood. In primary analyses, we examined average PM_2.5_ exposure during the 3rd trimester, average BC exposure during the 3rd trimester, traffic density based on address at delivery, and major roadway proximity based on address at delivery. In secondary analyses, we examined average PM_2.5_ and BC exposures during the 1st and 2nd trimesters. For all analyses, our outcomes included (1) BMI predicted by the cubic spline model at 6 month intervals, and (2) age of child (months), magnitude (kg/m^2^), and velocity (kg/m^2^/month) at BMI peak and BMI rebound. We also estimated the association of each exposure with overall BMI trajectory by including the exposures as fixed effects in the mixed-effects models. For analyses of BMI peak and rebound, we restricted the analyses to children with estimable BMI peak and rebound (*n* = 1,396–1,649).

To account for the exponential spatial decay of traffic pollution (27), we *a priori* categorized residential proximity to major roadway as > 200 m, 100 to < 200 m, 50 to < 100 m, and < 50 m, as we have done previously (11, 12). We initially modeled BC, PM_2.5_, and neighborhood traffic density in quartiles. We did not observe non-linearity in exposure–outcome relationships, and so we also modeled these variables as continuous measures, scaled by the interquartile range (IQR) of each exposure. We log-transformed neighborhood traffic density, which was right-skewed, using natural logarithms.

We first fit models adjusted only for child sex, followed by full multivariable models for each exposure–outcome relationship. We included additional covariates potentially associated with air pollution exposure and/or childhood growth: maternal age (continuous), education (with or without college degree), smoking habits (smoked during pregnancy, formerly smoked, never smoked), and parity (nulliparous or multiparous); child race/ethnicity (white, black, Hispanic, Asian or other); and census tract median household income (continuous) and percent below poverty (continuous). To account for trends in traffic pollution and growth by season and over time, we also included season (continuous sine and cosine of date) and date (continuous) of birth in multivariable models. We did not include gestational weight gain, maternal glucose tolerance, or gestational age in our models because these variables may be on the causal pathway, and their inclusion could introduce collider bias (28). We substituted maternal for child race/ethnicity in 10% of participants missing data on child race/ethnicity. Between 98.7 and 99.5% of participants had complete covariate information for the multivariable models. We assessed for effect modification by child sex, based on prior data suggesting the possibility of sex-specific associations in relation to prenatal air pollution exposure (29, 30). We found no effect modification, so we present all results without stratification or inclusion of an interaction term for child sex.

We used Stata 15 (StataCorp LP, Texas, USA) for all analyses.

## Results

### Population Characteristics

Sixty six percent of mothers were college graduates, 69% were non-smokers, and 52% were nulliparous. 64% of children were white. Third trimester median (IQR, range) PM_2.5_ concentration was 11.4 (1.7, 7.7–17.3) μg/m^3^ which is below the Environmental Protection Agency air quality standard for annual PM_2.5_ exposure (15 μg/m^3^ at the time of the study and 12 μg/m^3^ currently in 2018). Third trimester median (IQR, range) BC concentration was 0.7 (0.3, 0.1–1.6) μg/m^3^ which is consistent with the annual US urban average (ranged from 0.2 to 1.9 μg/m^3^) during 2005–2007 (31). At the time of delivery, median (IQR, range) neighborhood traffic density was 857 (1,452, 0–30,900) vehicles/day x km of road within 100 m of residential address; most mothers (88%) lived > 200 m from a major roadway, and 3% lived <50 m (Table 1). Correlations between exposures were moderate (Spearman correlation coefficients ranged from −0.37 to 0.51) and reported previously in detail in a similar subset of the Project Viva cohort (11).

**Table 1:** Characteristics of participants overall and by quartile of 3rd trimester PM_2.5_.

**PM_**2.5**_ (μg/m^**3**^), Max-Min**	**Overall** **(7.7–17.3)**	**Quartiles of PM**_****2.5****_			
		**Q1** **(7.7-10.5)**	**Q2** **(10.5–11.4)**	**Q3** **(11.4–12.2)**	**Q4** **(12.2–17.3)**
**MEAN (SD) OR %**					
**Maternal characteristics**					
Age at enrollment (years)	32.0 (5.2)	32.2 (4.6)	31.9 (5.3)	31.7 (5.4)	32.5 (5.3)
College graduate	66	72	67	66	63
Smoking habits					
Never	68	64	65	73	71
Prior to pregnancy	20	23	21	16	17
During pregnancy	12	13	13	10	12
Nulliparous	48	45	46	52	46
**Household characteristics**					
Census tract median income (US dollars/year)	57,921 (21,310)	64,794 (22,397)	60,855 (20,411)	53,698 (19,171)	52,423 (20,778)
Census tract% below poverty	10 (9)	7 (8)	8 (8)	11 (10)	12 (10)
**Child characteristics**					
Gestational age at delivery (weeks)	39.5 (1.8)	39.2 (2.0)	39.7 (1.6)	39.5 (1.9)	39.6 (1.5)
Female	49	47	48	50	51
Race/ethnicity					
White	64	73	69	59	57
Black	16	12	15	18	20
Hispanic	5	4	3	7	6
Asian	4	4	4	5	3
Other	10	8	8	10	13
**MEDIAN (IQR) OR %**					
**Traffic pollution**					
3rd trimester black carbon (μg/m^3^)	0.7 (0.3)	0.5 (0.2)	0.6 (0.3)	0.7 (0.3)	0.8 (0.3)
Neighborhood traffic density at delivery (km*vehicles/day)	857 (1,452)	531 (1,190)	658 (1,420)	1,016 (1,373)	1,223 (1,697)
Home proximity to major roadway at delivery					
< 50 m	3	3	3	4	5
50– < 100 m	3	1	3	3	4
100– < 200 m	6	3	7	9	7
≥200 m	88	93	87	84	84

Each child had a median [interquartile range (IQR)] of 13 (7) BMI measures between birth and mid-childhood. The mean (SD) of age at BMI peak was 8.4 (2.7) months and at BMI rebound was 59.4 (19.4) months. The mean (SD) of the magnitude of the BMI peak was 18.0 (1.4) kg/m^2^ and of the BMI rebound was 16.0 (1.2) kg/m^2^. The mean (SD) of velocity to BMI peak was 0.5 (0.2) kg/m^2^/month and to BMI rebound was −0.04 (0.02) kg/m^2^/month. As compared to boys, girls were older at peak BMI (8.9 vs. 8.1 months), younger at BMI rebound (55.5 vs. 63.1 months), had a lower magnitude at BMI peak (17.7 vs. 18.3 kg/m^2^) and rebound (15.9 vs. 16.1 kg/m^2^), and had a slower velocity to BMI peak (0.5 vs. 0.6 kg/m^2^/month).

### Traffic Pollution Exposure and Childhood BMI Trajectory

**Figure 1:**
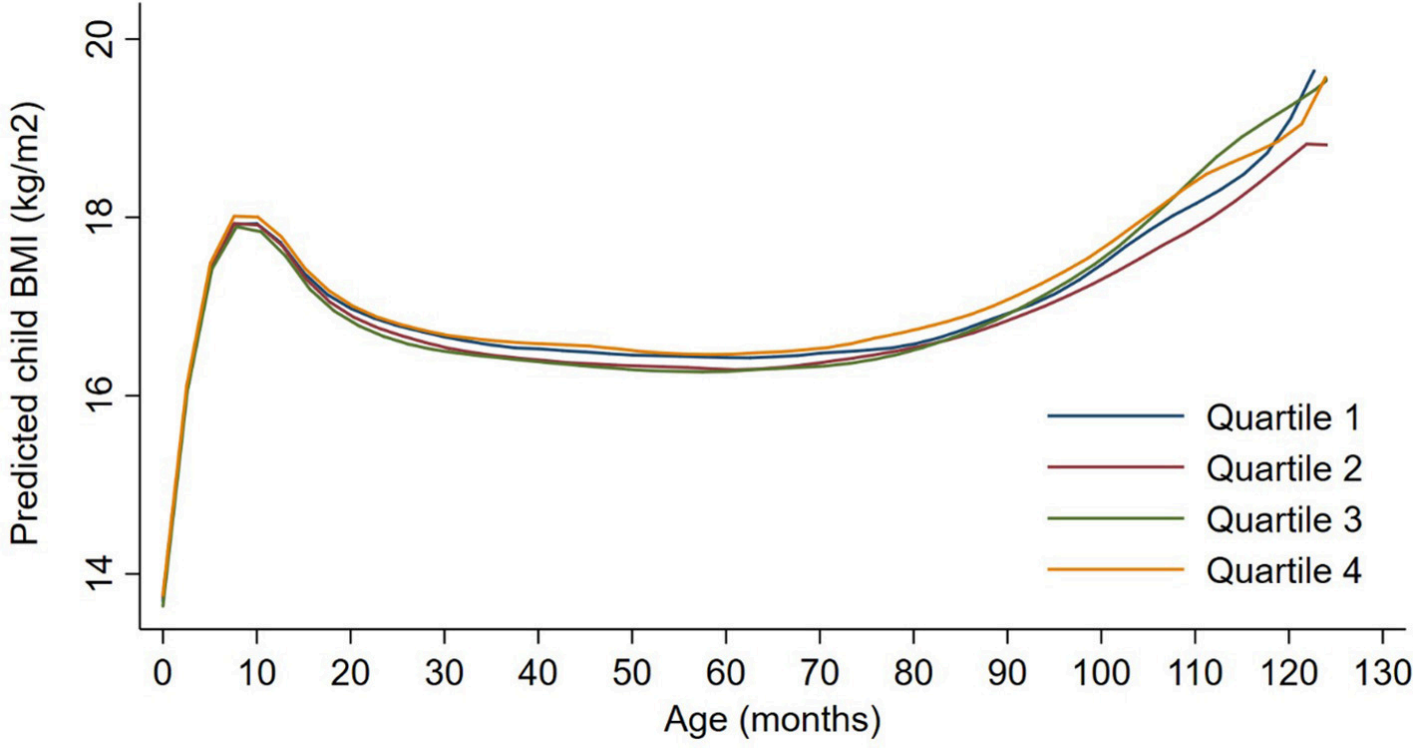
Child BMI trajectories from birth to mid-childhood according to quartiles of 3rd trimester PM_2.5_ exposure. Trajectories were additionally adjusted for date of birth, sine/cosine of the date of birth, maternal age, educational attainment, parity, smoking history, median household income, census tract% below poverty, child sex, and race/ethnicity.

**Figure 2:**
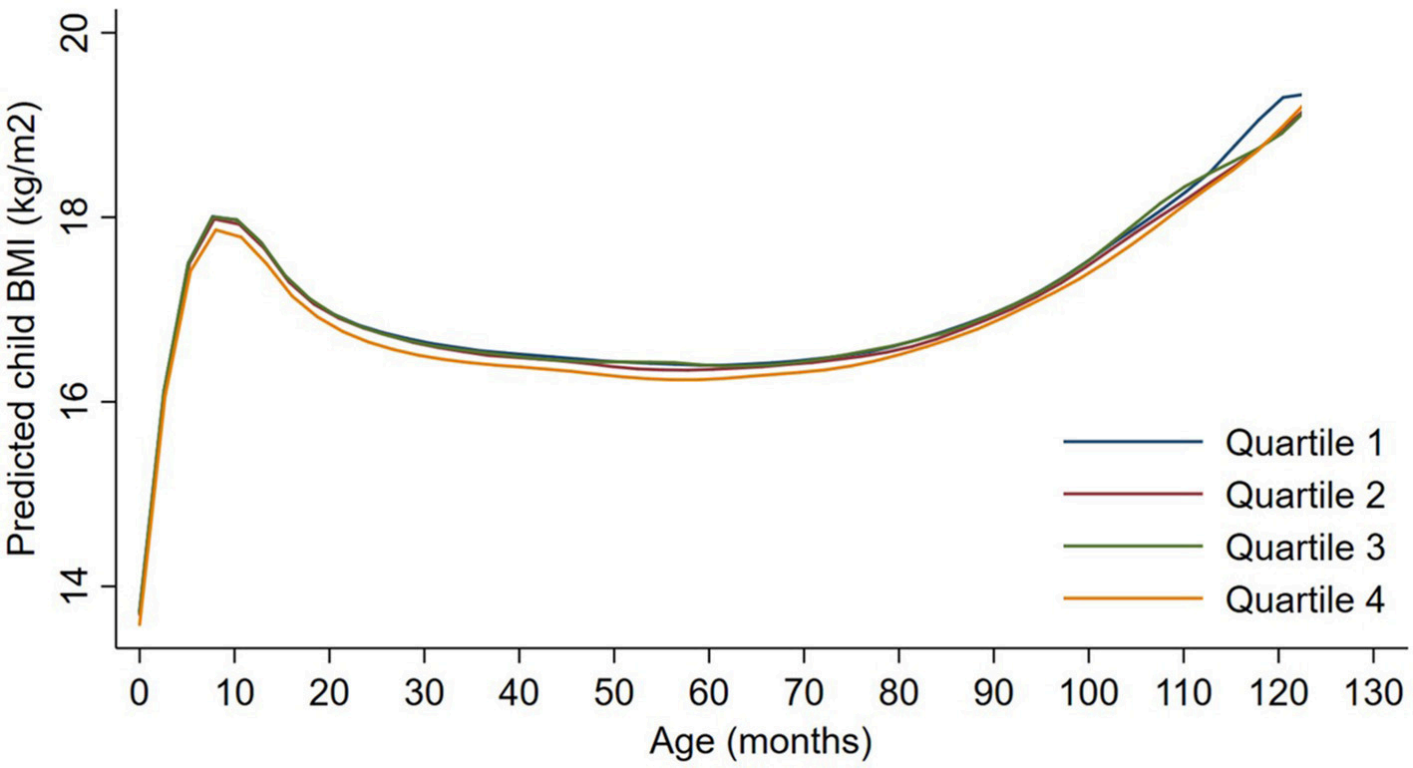
Child BMI trajectories from birth to mid-childhood according to quartiles of 3rd trimester black carbon exposure. Trajectories were additionally adjusted for date of birth, sine/cosine of the date of birth, maternal age, educational attainment, parity, smoking history, median household income, census tract% below poverty, child sex, and race/ethnicity.

**Figure 3:**
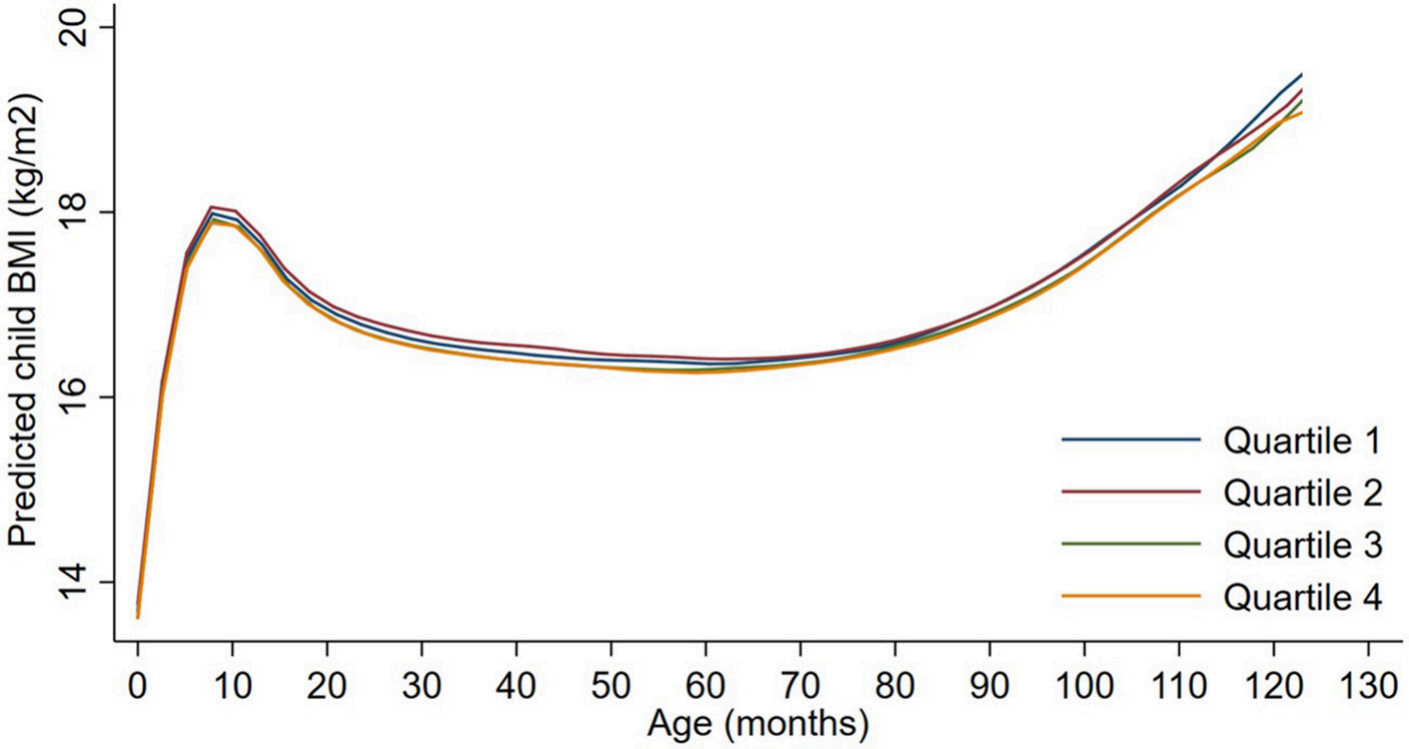
Child BMI trajectories from birth to mid-childhood according to quartiles of ln-transformed neighborhood traffic density. Trajectories were additionally adjusted for date of birth, sine/cosine of the date of birth, maternal age, educational attainment, parity, smoking history, median household income, census tract% below poverty, child sex and, race/ethnicity.

**Figure 4:**
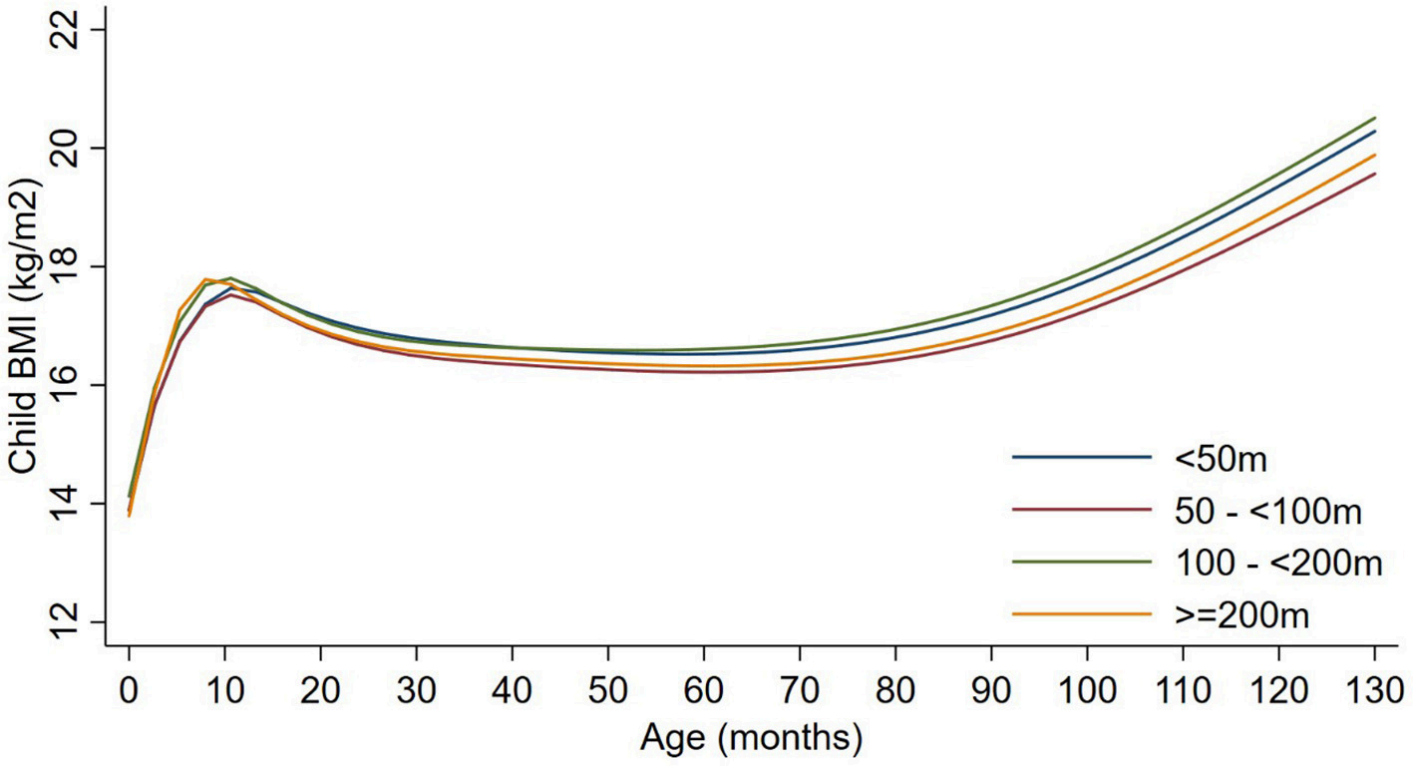
Child BMI trajectories from birth to mid-childhood according to distance to nearest major roadway. Trajectories were additionally adjusted for date of birth, sine/cosine of the date of birth, maternal age, educational attainment, parity, smoking history, median household income, census tract% below poverty, child sex, and race/ethnicity.

Exposures to PM_2.5_, BC, neighborhood traffic density, and home roadway proximity in late pregnancy were not associated with any of the BMI outcomes at any age in sex-adjusted or covariate-adjusted models, regardless of whether the exposure was represented in quartiles (Figures 1–3), per IQR increment (Supplemental Tables 2–4), or by category of roadway proximity (Figure 4; Supplemental Table 5). Effect estimates were in the hypothesized direction for neighborhood traffic density and home roadway proximity, although confidence intervals crossed the null and estimates were quite small in magnitude. For example, each IQR increment greater natural log-transformed neighborhood traffic density was associated with a 0.01 (95% CI: −0.06, 0.04) kg/m^2^ lower BMI at birth, a peak BMI 0.05 (−0.03, 0.14) kg/m^2^ higher, and a higher BMI throughout childhood, with the strongest associations with childhood BMI at 12–18 months of age [e.g., 0.05 (-0.03, 0.13) kg/m^2^ higher BMI per IQR increment neighborhood traffic density at 12 months of age]. The associations of home roadway proximity and childhood BMI trajectory were also in the hypothesized direction, with stronger associations as children approached 120 months (10 years) of age, although associations were non-monotonic and confidence intervals consistently crossed the null. For example, children of mothers who lived closest (< 50 m) vs. farthest (>200 m) from the nearest major roadway had a BMI that was 0.46 (−0.21, 0.59) kg/m^2^ higher at 120 months of age.

In secondary analyses, 1st and 2nd trimester residential PM_2.5_ and BC exposures were not associated with BMI trajectory (data not shown), and directionality and magnitude of effect estimates were similar to 3rd trimester exposures.

## Discussion

In a large, prospective Massachusetts pre-birth cohort, prenatal exposures to PM_2.5_ and BC were not related to BMI trajectory. Neighborhood traffic density and home roadway proximity were associated with lower BMI at birth and greater gains in BMI throughout childhood, but effect sizes were small and confidence intervals consistently crossed the null.

In the present study, we leveraged clinical and research data to estimate BMI trajectories for each participant from birth through mid-childhood. Our findings are partly consistent with previously reported observations in this cohort of prenatal traffic pollution on discrete research measures of growth and adiposity. We previously observed late prenatal exposures to BC, neighborhood traffic density, and home roadway proximity to be associated with lower birth weight-for-gestational age z-score, greater neighborhood traffic density to be associated with more rapid weight-for-length (WFL) gain in infancy (12), and home roadway proximity to be associated with greater BMI z-score in early childhood and higher central and total adiposity in early and mid-childhood (11).

We are aware of only two cohorts besides ours that have examined prenatal air pollution exposure and growth/adiposity in childhood (13, 14). The Boston-based Asthma Coalition on Community, Environment, and Social Stress (ACCESS) cohort (*n* = 239) observed associations of prenatal residential PM_2.5_ exposure with BMI z-score and total fat mass at 4 years of age in boys only (13). The Boston Birth Cohort (BBC) (*n* = 1,446) observed associations between prenatal residential PM_2.5_ exposure and greater odds of overweight or obesity as assessed at each participant's last recorded well-child check from 2 to 9 years of age (14). In both the ACCESS cohort and the BBC, associations with child adiposity were most pronounced for PM_2.5_ exposures during the 2nd trimester. We previously reported associations of prenatal PM_2.5_ exposure with early and mid-childhood BMI to be null regardless of trimester of exposure (11), and in the present study, prenatal residential PM_2.5_ exposure in any trimester was similarly not associated with childhood BMI trajectory. As compared to Project Viva, both the ACCESS cohort and the BBC comprise a greater number of children of lower socioeconomic status with obese mothers, factors which may increase susceptibility to prenatal air pollution exposure. Consistent with this observation, the BBC showed the strongest associations between prenatal air pollution exposure and child overweight/obesity in children of obese mothers (14). Furthermore, rates of breastfeeding in Project Viva (88%) (32) were higher than in the ACCESS cohort (68%) (33) or BBC (64%) (14), and recent studies suggest that breastfeeding may protect against PM_2.5_-induced health effects (34, 35). Thus, while our null findings are in contrast to studies of prenatal air pollution and child adiposity published in two other Boston cohorts, this discrepancy may be a result of differences in study population.

The present study is the first of which we are aware to examine prenatal air pollution exposure in relation to BMI trajectory across childhood. Examining a BMI trajectory outcome is advantageous because it flexibly allows identification of the time window during childhood when growth may be most affected by prenatal exposures. We found no association between prenatal exposure to traffic pollution and BMI estimated at multiple timepoints through 10 years of age, and we also found no association with overall BMI trajectory or with the timing or magnitude of BMI peak or rebound, which are known to predict greater susceptibility to cardio-metabolic disease later in life (36, 37). Additional studies of prenatal air pollution exposure and child growth trajectory, particularly in cohorts of children potentially more susceptible to air pollution exposure based on socioeconomic status or maternal weight, will help to further elucidate this potential association.

Our cohort is the first, so far as we know, to examine prenatal exposure to markers of traffic pollution other than regional PM_2.5_ in relation to growth in later childhood. Although associations did not reach statistical significance, we found neighborhood traffic density and home roadway proximity to be associated with lower BMI at birth, higher peak BMI in infancy, and a higher BMI throughout childhood—the directionalities expected based on our *a priori* hypotheses. As we have noted previously (11), the possible stronger impact of roadway/traffic as compared to PM_2.5_ or BC on child growth and adiposity in our cohort may be explained by independent associations of noise (38), light (39), ultrafine particles (40), or other roadway features distinct from air pollution or from the pollutants we measured, with child growth.

A strength of our study is that it is the largest prospective cohort to date that has analyzed prenatal air pollution exposure and growth in later childhood. In addition, we employ a growth trajectory approach and examine multiple pollutants. Limitations that may have prevented us from observing an association between prenatal air pollution exposure and growth in later childhood are lack of information on maternal time-activity patterns, a relatively high socioeconomic status cohort with a lower incidence of maternal overweight compared to previously published studies, and generally low levels of air pollution. Also, we assessed maternal smoking by questionnaire but did not biochemically validate the exposure. In addition, we did not have data to evaluate repeated measures of body composition, and we predicted BMI trajectories from statistical models rather than directly measuring BMI at each time point, but our models are precise as evidenced by mean residual errors (differences between observed and predicted BMI) close to zero across all ages (26).

In summary, neighborhood traffic density and home roadway proximity were associated with lower BMI at birth and greater gains in BMI throughout childhood, but observed effects were small and confidence intervals consistently crossed the null. We found no association between residential PM_2.5_ or BC exposure and childhood BMI trajectory. Thus, while there may be a role for roadway features distinct from the pollutants we measured, we found no persistent effect of prenatal traffic pollution on childhood BMI trajectory in a population exposed to modest levels of air pollution.

## Data Availability Statement

Policies for using Project Viva data are publicly available online at: https://www.hms.harvard.edu/viva/policies-for-using-our-data.pdf.

## Author Contributions

AF, BC, DG, and EO conceived this analysis. JS, PK, IK, HL-G, and DG developed air pollution models and/or applied them to this cohort. IA and SR-S performed the analysis. AF drafted the manuscript. All authors critically reviewed the manuscript.

### Conflict of Interest Statement

The authors declare that the research was conducted in the absence of any commercial or financial relationships that could be construed as a potential conflict of interest.
