# Supplementary material for: Prenatal Exposure to Traffic Pollution and Childhood Body Mass Index Trajectory
Source: Front Endocrinol (Lausanne). 2019 Jan 7;9:771. doi: 10.3389/fendo.2018.00771 (PMC6330299; doi:10.3389/fendo.2018.00771)
Supplement: Supplementary file 1 [file Table_1.DOCX]

Supplementary Material

**Prenatal Exposure to Traffic Pollution and Childhood Body Mass Index Trajectory**

Abby F. Fleisch^*^, Izzuddin M. Aris, Sheryl L. Rifas-Shiman, Brent A. Coull, Heike Luttmann-Gibson, Petros Koutrakis, Joel D. Schwartz, Itai Kloog, Diane R. Gold, Emily Oken

*Correspondence: Abby F. Fleisch: afleisch@mmc.org

**Supplemental Table 1**: Characteristics of Project Viva participants (n=2,128) included and

excluded from this analysis

|  | | **Included**  **(n=1649)** |  | **Excluded**  **(n=479)** |  |
| --- | --- | --- | --- | --- | --- |
|  |  |  |  |  |  |
|  | | **Mean (SD) or %** | | |  |
| **Maternal characteristics** | |  |  |  |  |
| **Age at enrollment (years)** | | 32.0 (5.2) |  | 31.1 (5.2) |  |
| **College graduate** | | 66 |  | 60 |  |
| **Smoking habits** | |  |  |  |  |
|  | Never | 68 |  | 69 |  |
|  | Prior to pregnancy | 20 |  | 17 |  |
|  | During pregnancy | 12 |  | 15 |  |
| **Nulliparous** | | 48 |  | 48 |  |
|  | |  |  |  |  |
| **Child characteristics** | |  |  |  |  |
| **Gestational age at delivery (weeks)** | | 39.5 (1.8) |  | 39.2 (2.4) |  |
| **Female** | | 49 |  | 48 |  |
| **Race/ethnicity** | |  |  |  |  |
|  | White | 64 |  | 62 |  |
|  | Black | 16 |  | 18 |  |
|  | Hispanic | 5 |  | 10 |  |
|  | Asian | 4 |  | 7 |  |
|  | Other | 10 |  | 4 |  |
|  |  |  |  |  |  |
| **Household characteristics** | |  |  |  |  |
| **Census tract median household income (US dollars/year)** | | 57,168 (21,256) |  | 56,444 (21,772) |  |
| **Census tract % below poverty** | | 9.9 (9.1) |  | 10.1 (9.0) |  |

**Supplemental Table 2**: Associations of 3^rd^-trimester PM_2.5_ exposure (per IQR increment) with child BMI parameters from birth to mid-childhood

|  |  | **Sex-adjusted** | | |  | **Fully-adjusted^a^** | | |
| --- | --- | --- | --- | --- | --- | --- | --- | --- |
|  |  | **β** | **95% CI** | |  | **β** | **95% CI** | |
|  |  |  | **Low** | **High** |  |  | **Low** | **High** |
| **BMI peak parameters (n=1396)** | |  |  |  |  |  |  |  |
|  | Age (months) | 0.01 | -0.17 | 0.18 |  | 0.05 | -0.15 | 0.25 |
|  | Magnitude (kg/m^2^) | 0.05 | -0.04 | 0.14 |  | 0.02 | -0.08 | 0.12 |
|  | Velocity (kg/m^2^/month) | 0.00 | -0.01 | 0.01 |  | 0.00 | -0.02 | 0.01 |
| **BMI rebound parameters (n=1396)** | |  |  |  |  |  |  |  |
|  | Age (months) | 0.59 | -0.67 | 1.84 |  | 1.21 | -0.19 | 2.61 |
|  | Magnitude (kg/m^2^) | 0.02 | -0.06 | 0.10 |  | -0.01 | -0.10 | 0.07 |
|  | Velocity (kg/m^2^/month) | 0.00 | -0.002 | 0.001 |  | 0.00 | -0.001 | 0.002 |
| **Predicted BMI (kg/m^2^; n=1456)** | |  |  |  |  |  |  |  |
|  | Birth | 0.02 | -0.03 | 0.07 |  | 0.02 | -0.03 | 0.08 |
|  | 6 months | 0.04 | -0.05 | 0.12 |  | 0.001 | -0.10 | 0.10 |
|  | 12 months | 0.04 | -0.05 | 0.12 |  | 0.002 | -0.09 | 0.09 |
|  | 18 months | 0.03 | -0.04 | 0.11 |  | 0.002 | -0.09 | 0.09 |
|  | 24 months | 0.03 | -0.05 | 0.11 |  | -0.001 | -0.09 | 0.09 |
|  | 30 months | 0.03 | -0.05 | 0.11 |  | -0.01 | -0.10 | 0.08 |
|  | 36 months | 0.04 | -0.05 | 0.12 |  | -0.01 | -0.11 | 0.08 |
|  | 42 months | 0.04 | -0.05 | 0.12 |  | -0.02 | -0.12 | 0.08 |
|  | 48 months | 0.04 | -0.05 | 0.13 |  | -0.03 | -0.13 | 0.08 |
|  | 54 months | 0.04 | -0.06 | 0.14 |  | -0.03 | -0.14 | 0.08 |
|  | 60 months | 0.04 | -0.06 | 0.15 |  | -0.04 | -0.15 | 0.08 |
|  | 66 months | 0.05 | -0.07 | 0.16 |  | -0.04 | -0.17 | 0.08 |
|  | 72 months | 0.05 | -0.07 | 0.17 |  | -0.05 | -0.18 | 0.08 |
|  | 78 months | 0.05 | -0.08 | 0.18 |  | -0.06 | -0.20 | 0.09 |
|  | 84 months | 0.05 | -0.08 | 0.19 |  | -0.06 | -0.21 | 0.09 |
|  | 90 months | 0.06 | -0.09 | 0.20 |  | -0.07 | -0.23 | 0.10 |
|  | 96 months | 0.06 | -0.10 | 0.22 |  | -0.07 | -0.24 | 0.10 |
|  | 102 months | 0.06 | -0.11 | 0.23 |  | -0.08 | -0.26 | 0.11 |
|  | 108 months | 0.07 | -0.11 | 0.25 |  | -0.08 | -0.28 | 0.12 |
|  | 114 months | 0.07 | -0.12 | 0.26 |  | -0.08 | -0.30 | 0.13 |
|  | 120 months | 0.08 | -0.13 | 0.28 |  | -0.09 | -0.31 | 0.14 |
| **Overall BMI trajectory (kg/m^2^; n=1456)** | | 0.03 | -0.04 | 0.10 |  | 0.02 | -0.06 | 0.09 |

^a^ Adjusted for date of birth, sine/cosine of the date of birth, maternal age, educational attainment, parity, smoking history, median household income, census tract % below poverty, child sex and race/ethnicity

**Supplemental Table 3**: Associations of 3^rd^-trimester black carbon exposure (per IQR increment) with child BMI parameters from birth to mid-childhood

|  |  | **Sex-adjusted** | | |  | **Fully-adjusted** | | |
| --- | --- | --- | --- | --- | --- | --- | --- | --- |
|  |  | **β** | **95% CI** | |  | **β** | **95% CI** | |
|  |  |  | **Low** | **High** |  |  | **Low** | **High** |
| **BMI peak parameters (n=1562)** | |  |  |  |  |  |  |  |
|  | Age (months) | -0.06 | -0.24 | 0.12 |  | 0.03 | -0.21 | 0.27 |
|  | Magnitude (kg/m^2^) | 0.02 | -0.07 | 0.12 |  | -0.05 | -0.17 | 0.08 |
|  | Velocity (kg/m^2^/month) | 0.01 | 0.00 | 0.02 |  | -0.004 | -0.02 | 0.01 |
| **BMI rebound parameters (n=1562)** | |  |  |  |  |  |  |  |
|  | Age (months) | **-1.34** | **-2.65** | **-0.02** |  | 0.08 | -1.62 | 1.79 |
|  | Magnitude (kg/m^2^) | 0.03 | -0.06 | 0.11 |  | -0.04 | -0.15 | 0.07 |
|  | Velocity (kg/m^2^/month) | -0.002 | -0.003 | 0.00 |  | 0.00 | -0.003 | 0.002 |
| **Predicted BMI (kg/m^2^; n=1633)** | |  |  |  |  |  |  |  |
|  | Birth | **-0.05** | **-0.11** | **0.00** |  | -0.01 | -0.08 | 0.06 |
|  | 6 months | 0.02 | -0.07 | 0.11 |  | -0.04 | -0.16 | 0.08 |
|  | 12 months | 0.02 | -0.07 | 0.11 |  | -0.04 | -0.15 | 0.07 |
|  | 18 months | 0.01 | -0.08 | 0.09 |  | -0.04 | -0.15 | 0.07 |
|  | 24 months | 0.00 | -0.09 | 0.08 |  | -0.04 | -0.15 | 0.06 |
|  | 30 months | -0.01 | -0.10 | 0.07 |  | -0.05 | -0.16 | 0.06 |
|  | 36 months | -0.02 | -0.10 | 0.07 |  | -0.05 | -0.16 | 0.06 |
|  | 42 months | -0.01 | -0.10 | 0.08 |  | -0.05 | -0.17 | 0.07 |
|  | 48 months | 0.00 | -0.10 | 0.09 |  | -0.05 | -0.18 | 0.07 |
|  | 54 months | 0.01 | -0.09 | 0.11 |  | -0.05 | -0.18 | 0.08 |
|  | 60 months | 0.03 | -0.08 | 0.14 |  | -0.04 | -0.18 | 0.10 |
|  | 66 months | 0.05 | -0.07 | 0.16 |  | -0.04 | -0.19 | 0.11 |
|  | 72 months | 0.07 | -0.05 | 0.19 |  | -0.03 | -0.19 | 0.13 |
|  | 78 months | 0.09 | -0.04 | 0.23 |  | -0.03 | -0.20 | 0.15 |
|  | 84 months | 0.12 | -0.03 | 0.26 |  | -0.02 | -0.21 | 0.16 |
|  | 90 months | 0.14 | -0.02 | 0.29 |  | -0.02 | -0.22 | 0.18 |
|  | 96 months | 0.16 | -0.01 | 0.32 |  | -0.02 | -0.23 | 0.20 |
|  | 102 months | 0.17 | -0.01 | 0.35 |  | -0.01 | -0.24 | 0.21 |
|  | 108 months | 0.19 | -0.003 | 0.38 |  | -0.01 | -0.26 | 0.23 |
|  | 114 months | 0.20 | -0.004 | 0.40 |  | -0.02 | -0.28 | 0.24 |
|  | 120 months | 0.21 | -0.01 | 0.43 |  | -0.02 | -0.30 | 0.26 |
| **Overall BMI trajectory (kg/m^2^; n=1633)** | | -0.05 | -0.13 | 0.02 |  | -0.04 | -0.13 | 0.06 |

^a^ Adjusted for date of birth, sine/cosine of the date of birth, maternal age, educational attainment, parity, smoking history, median household income, census tract % below poverty, child sex and race/ethnicity

**Supplemental Table 4**: Associations of ln-transformed neighbourhood traffic density (per IQR increment) with child BMI parameters from birth to mid-childhood

|  |  | **Sex-adjusted** | | |  | **Fully-adjusted^a^** | | |
| --- | --- | --- | --- | --- | --- | --- | --- | --- |
|  |  | **β** | **95% CI** | |  | **β** | **95% CI** | |
|  |  |  | **Low** | **High** |  |  | **Low** | **High** |
| **BMI peak parameters (n=1555)** | |  |  |  |  |  |  |  |
|  | Age (months) | 0.02 | -0.13 | 0.18 |  | 0.06 | -0.11 | 0.22 |
|  | Magnitude (kg/m^2^) | 0.06 | -0.02 | 0.14 |  | 0.05 | -0.03 | 0.14 |
|  | Velocity (kg/m^2^/month) | 0.01 | -0.001 | 0.02 |  | 0.005 | -0.01 | 0.02 |
| **BMI rebound parameters (n=1555)** | |  |  |  |  |  |  |  |
|  | Age (months) | -0.03 | -1.15 | 1.08 |  | 0.58 | -0.60 | 1.77 |
|  | Magnitude (kg/m^2^) | 0.03 | -0.04 | 0.10 |  | 0.02 | -0.06 | 0.09 |
|  | Velocity (kg/m^2^/month) | 0.00 | -0.002 | 0.001 |  | 0.00 | -0.001 | 0.002 |
| **Predicted BMI (kg/m^2^; n=1625)** | |  |  |  |  |  |  |  |
|  | Birth | -0.04 | -0.08 | 0.01 |  | -0.01 | -0.06 | 0.04 |
|  | 6 months | 0.04 | -0.04 | 0.12 |  | 0.04 | -0.04 | 0.12 |
|  | 12 months | 0.05 | -0.03 | 0.12 |  | 0.05 | -0.03 | 0.13 |
|  | 18 months | 0.04 | -0.03 | 0.11 |  | 0.04 | -0.03 | 0.12 |
|  | 24 months | 0.03 | -0.04 | 0.10 |  | 0.04 | -0.04 | 0.11 |
|  | 30 months | 0.02 | -0.05 | 0.10 |  | 0.03 | -0.04 | 0.11 |
|  | 36 months | 0.02 | -0.05 | 0.09 |  | 0.03 | -0.05 | 0.10 |
|  | 42 months | 0.02 | -0.06 | 0.10 |  | 0.02 | -0.06 | 0.10 |
|  | 48 months | 0.02 | -0.06 | 0.10 |  | 0.02 | -0.07 | 0.11 |
|  | 54 months | 0.03 | -0.06 | 0.11 |  | 0.02 | -0.07 | 0.11 |
|  | 60 months | 0.04 | -0.06 | 0.13 |  | 0.02 | -0.08 | 0.12 |
|  | 66 months | 0.04 | -0.05 | 0.14 |  | 0.02 | -0.08 | 0.13 |
|  | 72 months | 0.05 | -0.05 | 0.16 |  | 0.02 | -0.09 | 0.14 |
|  | 78 months | 0.06 | -0.05 | 0.18 |  | 0.03 | -0.09 | 0.14 |
|  | 84 months | 0.07 | -0.05 | 0.20 |  | 0.03 | -0.10 | 0.15 |
|  | 90 months | 0.08 | -0.05 | 0.21 |  | 0.03 | -0.11 | 0.16 |
|  | 96 months | 0.09 | -0.05 | 0.23 |  | 0.02 | -0.12 | 0.17 |
|  | 102 months | 0.09 | -0.06 | 0.25 |  | 0.02 | -0.14 | 0.18 |
|  | 108 months | 0.10 | -0.06 | 0.26 |  | 0.02 | -0.15 | 0.18 |
|  | 114 months | 0.10 | -0.07 | 0.27 |  | 0.01 | -0.17 | 0.19 |
|  | 120 months | 0.10 | -0.08 | 0.29 |  | 0.003 | -0.19 | 0.20 |
| **Overall BMI trajectory (kg/m^2^; n=1625)** | | -0.02 | 0.08 | 0.04 |  | 0.005 | -0.06 | 0.07 |

^a^ Adjusted for date of birth, sine/cosine of the date of birth, maternal age, educational attainment, parity, smoking history, median household income, census tract % below poverty, child sex and race/ethnicity

**Supplemental Table 5**: Associations of home proximity to major roadway (<50m, 50-<100m, 100-<200m or ≥200m) with child BMI parameters from birth to mid-childhood

|  |  | **Sex-adjusted [β (95% CI)]** | | | |  | **Fully-adjusted^a^ [β (95% CI)]** | | | |
| --- | --- | --- | --- | --- | --- | --- | --- | --- | --- | --- |
|  |  | **<50m** | **50-<100m** | **100-<200m** | **≥200m** |  | **<50m** | **50-<100m** | **100-<200m** | **≥200m** |
| **BMI peak parameters (n=1578)** | |  |  |  |  |  |  |  |  |  |
|  | Age (months) | 0.27  (-0.46,1.00) | -0.24  (-1.01,0.52) | -0.29  (-0.84,0.25) | ref |  | 0.30  (-0.44,1.04) | -0.19  (-0.97,0.59) | -0.28  (-0.83,0.28) | ref |
|  | Magnitude (kg/m^2^) | 0.07  (-0.31,0.45) | 0.04  (-0.36,0.44) | **0.32**  **(0.04,0.61)** | ref |  | 0.09  (-0.29,0.47) | 0.03  (-0.37,0.43) | **0.34**  **(0.05,0.63)** | ref |
|  | Velocity (kg/m^2^/month) | 0.03  (-0.02,0.07) | 0.03  (-0.02,0.08) | 0.03  (-0.01,0.06) | ref |  | 0.03  (-0.02,0.07) | 0.03  (-0.02,0.08) | 0.03  (-0.01,0.06) | ref |
| **BMI rebound parameters (n=1578)** | |  |  |  |  |  |  |  |  |  |
|  | Age (months) | 1.74  (-3.54,7.02) | -0.61  (-6.15,4.94) | -3.61  (-7.56,0.34) | ref |  | 1.25  (-4.04,6.54) | -1.24  (-6.80,4.33) | -3.89  (-7.87,0.09) | ref |
|  | Magnitude (kg/m^2^) | 0.02  (-0.31,0.36) | -0.05  (-0.40,0.31) | **0.28**  **(0.03,0.53)** | ref |  | 0.07  (-0.26,0.41) | -0.01  (-0.36,0.34) | **0.33**  **(0.07,0.58)** | ref |
|  | Velocity (kg/m^2^/month) | 0.003  (-0.003,0.01) | -0.003  (-0.01,0.004) | -0.004  (-0.01,0.0004) | ref |  | 0.003  (-0.004,0.01) | -0.004  (-0.01,0.003) | -0.005  (-0.01,0.004) | ref |
| **Predicted BMI (kg/m^2^; n=1649)** | |  |  |  |  |  |  |  |  |  |
|  | Birth | -0.21  (-0.43,0.02) | -0.14  (-0.38,0.09) | 0.18  (0.01,0.35) | ref |  | -0.17  (-0.40,0.05) | -0.17  (-0.41,0.06) | 0.19  (0.02,0.36) | ref |
|  | 6 months | 0.03  (-0.34,0.40) | 0.03  (-0.36,0.42) | 0.27  (-0.005,0.55) | ref |  | 0.05  (-0.33,0.42) | 0.02  (-0.38,0.41) | **0.29**  **(0.007,0.57)** | ref |
|  | 12 months | 0.14  (-0.20,0.49) | 0.008  (-0.36,0.38) | 0.23  (-0.03,0.48) | ref |  | 0.17  (-0.18,0.52) | 0.01  (-0.36,0.38) | 0.25  (-0.01,0.51) | ref |
|  | 18 months | 0.20  (-0.13,0.53) | -0.02  (-0.37,0.33) | 0.19  (-0.06,0.44) | ref |  | 0.23  (-0.10,0.57) | -0.005  (-0.36,0.35) | 0.22  (-0.03,0.47) | ref |
|  | 24 months | 0.22  (-0.11,0.55) | -0.04  (-0.39,0.31) | 0.18  (-0.07,0.42) | ref |  | 0.27  (-0.06,0.60) | -0.01  (-0.36,0.34) | 0.22  (-0.03,0.47) | ref |
|  | 30 months | 0.22  (-0.11,0.55) | -0.05  (-0.40,0.30) | 0.18  (-0.06,0.43) | ref |  | 0.28  (-0.06,0.61) | -0.008  (-0.36,0.34) | 0.23  (-0.02,0.48) | ref |
|  | 36 months | 0.21  (-0.14,0.55) | -0.06  (-0.42,0.31) | 0.20  (-0.06,0.46) | ref |  | 0.27  (-0.07,0.62) | -0.005  (-0.37,0.36) | 0.25  (-0.008,0.51) | ref |
|  | 42 months | 0.20  (-0.16,0.56) | -0.06  (-0.44,0.32) | 0.22  (-0.05,0.49) | ref |  | 0.27  (-0.09,0.63) | -0.005  (-0.39,0.38) | 0.27  (0.002,0.55) | ref |
|  | 48 months | 0.19  (-0.19,0.58) | -0.07  (-0.47,0.34) | 0.24  (-0.04,0.53) | ref |  | 0.28  (-0.11,0.66) | -0.008  (-0.41,0.40) | 0.30  (0.009,0.58) | ref |
|  | 54 months | 0.20  (-0.21,0.60) | -0.08  (-0.51,0.35) | 0.27  (-0.03,0.57) | ref |  | 0.28  (-0.12,0.69) | -0.02  (-0.44,0.41) | 0.32  (0.02,0.62) | ref |
|  | 60 months | 0.20  (-0.23,0.63) | -0.09  (-0.54,0.37) | 0.29  (-0.03,0.62) | ref |  | 0.29  (-0.14,0.72) | -0.03  (-0.48,0.43) | **0.34**  **(0.02,0.66)** | ref |
|  | 66 months | 0.21  (-0.25,0.67) | -0.10  (-0.59,0.39) | 0.32  (-0.02,0.66) | ref |  | 0.30  (-0.15,0.76) | -0.04  (-0.53,0.44) | **0.36**  **(0.02,0.71)** | ref |
|  | 72 months | 0.23  (-0.27,0.72) | -0.11  (-0.64,0.41) | 0.35  (-0.02,0.72) | ref |  | 0.32  (-0.17,0.81) | -0.06  (-0.58,0.46) | **0.39**  **(0.02,0.76)** | ref |
|  | 78 months | 0.24  (-0.29,0.77) | -0.13  (-0.69,0.43) | 0.37  (-0.02,0.77) | ref |  | 0.34  (-0.19,0.86) | -0.07  (-0.63,0.48) | **0.41**  **(0.02,0.81)** | ref |
|  | 84 months | 0.26  (-0.31,0.83) | -0.14  (-0.75,0.46) | 0.40  (-0.02,0.83) | ref |  | 0.35  (-0.21,0.92) | -0.09  (-0.69,0.51) | **0.43**  **(0.01,0.86)** | ref |
|  | 90 months | 0.28  (-0.34,0.89) | -0.16  (-0.81,0.49) | 0.43  (-0.03,0.89) | ref |  | 0.37  (-0.24,0.98) | -0.11  (-0.74,0.53) | **0.46**  **(0.003,0.91)** | ref |
|  | 96 months | 0.30  (-0.36,0.95) | -0.18  (-0.88,0.51) | 0.46  (-0.03,0.95) | ref |  | 0.39  (-0.26,1.04) | -0.13  (-0.82,0.56) | 0.48  (-0.005,0.97) | ref |
|  | 102 months | 0.31  (-0.39,1.02) | -0.20  (-0.95,0.54) | 0.49  (-0.03,1.02) | ref |  | 0.41  (-0.29,1.10) | -0.15  (-0.89,0.59) | 0.51  (-0.01,1.03) | ref |
|  | 108 months | 0.33  (-0.42,1.08) | -0.22  (-1.02,0.57) | 0.52  (-0.04,1.08) | ref |  | 0.42  (-0.32,1.17) | -0.17  (-0.96,0.62) | 0.54  (-0.02,1.09) | ref |
|  | 114 months | 0.35  (-0.45,1.16) | -0.25  (-1.10,0.61) | 0.55  (-0.05,1.15) | ref |  | 0.44  (-0.36,1.24) | -0.19  (-1.03,0.65) | 0.56  (-0.04,1.16) | ref |
|  | 120 months | 0.37  (-0.49,1.23) | -0.27  (-1.18,0.64) | 0.58  (-0.06,1.22) | ref |  | 0.46  (-0.39,1.31) | -0.21  (-1.11,0.69) | 0.59  (-0.05,1.23) | ref |
| **Overall BMI trajectory (kg/m^2^; n=1649)** | | -0.10  (-0.39,0.19) | -0.13  (-0.44,0.18) | **0.23**  **(0.02,0.44)** | ref |  | -0.05  (-0.34,0.24) | -0.15  (-0.45,0.16) | **0.26**  **(0.05,0.47)** | ref |

^a^ Adjusted for date of birth, sine/cosine of the date of birth, maternal age, educational attainment, parity, smoking history, median household income, census tract % below poverty, child sex and race/ethnicity
